# Cognition, educational attainment and diabetes distress predict poor health literacy in diabetes: A cross-sectional analysis of the SHELLED study

**DOI:** 10.1371/journal.pone.0267265

**Published:** 2022-04-20

**Authors:** Pamela Chen, Michele Callisaya, Karen Wills, Timothy Greenaway, Tania Winzenberg

**Affiliations:** 1 School of Medicine, University of Tasmania, Hobart, Australia; 2 Menzies Institute for Medical Research, University of Tasmania, Hobart, Australia; Freelance Consultant, Myanmar, MYANMAR

## Abstract

**Objectives:**

To identify factors that predict poor health literacy amongst people with diabetes.

**Design:**

Cross-sectional analysis of baseline data from a prospective study of diabetic foot disease.

**Setting:**

Patients attending a tertiary hospital diabetes outpatient clinic in Tasmania, Australia.

**Participants:**

222 people with diabetes mellitus, aged >40 years, with no history of foot ulceration, psychotic disorders or dementia.

**Outcome measures:**

Health literacy was measured using the short form Test of Functional Health Literacy in Adults (functional health literacy), and the Health Literacy Questionnaire (HLQ), which measures nine domains of health literacy. Predictors included demographic characteristics, cognition, diabetes distress, depression, and educational attainment.

**Results:**

In multivariable analysis, greater educational attainment (OR 0.88, 95% CI 0.76, 0.99) and poorer cognition (OR 0.71, 95% CI 0.63, 0.79) were associated with poorer functional health literacy. Age was negatively associated with domains of appraisal of health information and ability to find good health information (both beta = -0.01). Educational attainment was positively associated with four domains, namely having sufficient information to manage my health, actively managing my health, appraisal of and ability to find good health information (beta ranging from +0.03 to 0.04). Diabetes distress was negatively associated with five domains: having sufficient information to manage my health, social support for health, ability to actively engage with healthcare providers, navigating the healthcare system and ability to find good health information (beta ranging from -0.14 to -0.18).

**Conclusion:**

Poorer cognition and poorer educational attainment may be detrimental for an individual’s functional health literacy, and education, diabetes distress and older age detrimental across multiple health literacy domains. Clinicians and policy makers should be attuned to these factors when communicating with people with diabetes and in designing healthcare systems to be more health-literacy friendly in order to improve diabetes outcomes.

## Introduction

Health Literacy is defined by the World Health Organization as the “cognitive and social skills which determine the motivation and ability of individuals to gain access to, understand and use information in ways which promote and maintain good health” [1]. It comprises three domains—basic/functional literacy (basic reading and writing of health information), communicative literacy (more advanced cognitive skills required to extract and apply information to changing circumstances) and critical literacy (required to critically analyze information and to use it in decision making) [2]. Poor health literacy is a significant public health issue; up to 60 percent of adult Australians, and up to 85% of adults worldwide living with diabetes have low health literacy [3, 4], meaning they are likely to struggle, or not be able to effectively make healthcare decisions.

Health literacy is acknowledged as being critical for promoting good health practices and primary prevention of diseases. Associations have been reported between low health literacy and poorer use of preventative health services [5] and increased healthcare costs [6]. People with low health literacy are also more likely to misunderstand medication labels and make errors in taking their medications [5]. Amongst the elderly, people with low health literacy have greater all-cause mortality [7]. Therefore, healthcare professionals and systems need to be aware of health literacy requirements of their consumers and adequately tailor communication and resources to their needs.

People living with diabetes in particular have poor health literacy [8]. This is potentially a major problem, as diabetes is a demanding chronic condition requiring knowledge and skills in glucose management, administering medications as well as incorporating lifestyle changes to prevent complications. Understanding and applying written and verbal instructions and advice from health professionals can be complex, demanding and overwhelming for individuals. It is therefore no surprise that people with diabetes and lower health literacy are more likely to experience diabetes related complications such as cerebrovascular disease and retinopathy [9].

Despite the potential for poor health literacy to increase diabetic complications, few studies have explored predictors of inadequate health literacy amongst people with diabetes and they are limited by their focus on functional health literacy rather than its broader construct [10–12]. Factors with reported associations with low functional health literacy are gender, being of a nonwhite race [10], having a migrant background, lower education attainment [10, 11], and having a depressed mood [11], whereas better cognition was associated with higher functional health literacy [12]. Recognizing which socio-demographic factors predict poor health literacy across all its domains will enable health services to target health literacy deficits amongst particularly vulnerable patient groups. Therefore, the aim of this study was to identify predictors of functional and overall health literacy amongst people living with diabetes attending a tertiary outpatient clinic for diabetes care.

## Materials and methods

This is a cross-sectional analysis of baseline data from the Southern Tasmanian Health Literacy and Foot Ulcer Development in Diabetes Mellitus Study (SHELLED Study). SHELLED is a longitudinal study, with the primary aims of determining the associations of health literacy with risk factors for, and development of foot ulcers in people with diabetes over 4 years. Data collection processes and questionnaires used are described elsewhere [13]. In brief, participants were recruited from the Royal Hobart Hospital’s diabetes outpatient clinics. After providing informed consent, participants completed a series of questionnaires including two measures of health literacy and underwent an assessment of foot health at the Menzies Institute for Medical Research at the University of Tasmania.

### Ethics

SHELLED was approved by the Human Research Ethics Committee (Tasmania) Network (reference H0014284). All participants provided written consent prior to commencing study procedures.

### Measures

#### Health literacy

Health literacy was measured in two ways. The Short Form Test of Functional Health Literacy in Adults (S-TOFHLA) measured functional health literacy and the Health Literacy Questionnaire (HLQ) is a broader measure which was used to assess all health literacy domains [14].

The S-TOFHLA is a 36 item timed test of comprehension using a modified Cloze procedure, and requires participants to complete two passages, one concerning instructions to have an x-ray and another from the “patient rights and responsibilities” section of an American Medicaid application form [15]. Participants were advised of Australian equivalents of American terms, namely Medicaid (Medicare in Australia) and Temporary Assistance for Needy Families (TANF), which is similar to Centrelink in Australia. The possible score range for the S-TOFHLA is from 0 to 36. The S-TOFHLA has excellent reliability (Cronbach’s alpha 0.98) and validity (0.91) [15].

The HLQ comprises 9 scales assessing the following health literacy domains: 1. Feeling understood and supported by healthcare professionals (4 items); 2. Having sufficient information to manage my health (4 items); 3. Actively managing my health (5 items); 4. Social support for health (5 items); 5. Appraisal of health information (5 items); 6. Ability to actively engage with healthcare providers (5 items); 7. Navigating the health system (5 items); 8. Ability to find good health information (5 items); and 9. Understanding health information well enough to know what to do (5 items). Domains 1 to 5 are scored out of 4 (strongly disagree, disagree, agree, strongly agree). Domains 6 to 9, which measure difficulty of health-related tasks to the individual, are scored out of 5 (cannot do or always difficult, usually difficult, sometimes difficult, usually easy and always easy). Individual domains of the HLQ have good reliability with composite reliability ranging between 0.77 and 0.89 [14].

#### Other covariates

Sociodemographic variables assessed by questionnaire were age, gender, years of formal education, employment status, and annual household income. Participants reported their employment status as: 1. Employed part-time, 2. Employed full time, 3. Home duties, 4. Student, 5. Sole parent pension, 6. Disability pension, 7. Retired and 8. Unemployed. These categories were collapsed into 1. Employed full-time, 2. Employed part-time, 3. Not in paid employment (including categories 3–8 above). Annual household income had 4 categories (<$49,999, $50,000 to $99,999, >$100,000 and Rather not say), with “Rather not say” interpreted as missing data. Smoking status and medical history including of diabetes (type, age of diagnosis, monitoring of diabetes and insulin use) were also assessed by questionnaire.

Cognition was assessed using the Montreal Cognitive Assessment (MOCA) [16]. The MOCA is a validated screening tool with an internal consistency of 0.83 [16] and scores below 26/30 are considered indicative of cognitive impairment. Depression was measured using the Patient Health Questionnaire-9 (PHQ-9), which has a sensitivity and specificity of 92% and 82% respectively for the diagnosis of major depression according to the DSM-IV criteria [17]. The 17-item Diabetes Distress domain was used to measure diabetes-related distress [18]. Participants’ height and weight were measured using a stadiometer and calibrated domains respectively, and BMI calculated.

#### Statistics

Participant characteristics were summarized as mean (SD) for continuous variables and percentage (frequency) for categorical variables. The sample size of 220 was calculated based on the longitudinal component of this study [13]; required to detect differences in associations of S-TOFHLA categories with foot ulcer incidence over 4 years.

Functional and overall health literacy (HLQ) scores were analyzed separately. Functional health literacy is regularly categorized into adequate, marginal and inadequate respectively according to previously published cut-offs [19]. However these are arbitrary and population specific cut-offs [19] and in our sample S-TOFHLA scores were very unevenly distributed across these three categories. Therefore, we dichotomized our data into low (31 or less out of 36) and high functional health literacy (32 or more out of 36) which better represented the distribution of functional health literacy in our study population. As recommended, scores for individual domains on the HLQ were analyzed separately [14].

Associations with poor functional health literacy (S-TOFHLA categories) were estimated using logistic regression. Linear regression modelling was used to estimate associations of demographic variables with scores on each domain of the HLQ. Initial models were adjusted for age and gender and the final model determined using a purposeful covariate selection approach. Covariate selection was informed by clinical knowledge and the effect that different variables could have on health literacy, effect size and residual deviance of overall models. Adequacy of models were confirmed using the Hosmer-Lemeshow goodness of fit test.

As the number of individuals with missing data for relevant variables was very small (maximum of 2 people) [13], those with missing data required for each relevant regression analysis were excluded.

All analyses were performed using R.

#### Patient and public involvement

No patients were involved in setting the research question or the outcome measures, nor were any involved in developing plans for recruitment, design, or implementation of the study. No patients were asked to advise on interpretation or writing up of results.

## Results

### Participant characteristics

Of 411 people approached to participate, 222 consented and completed the study. We have previously published reasons for nonparticipation [13]. Characteristics of the 222 participants as a whole and in those with low vs high functional health literacy are given in Table 1.

**Table 1:** Demographic data for participants of the SHELLED study.

*Characteristic*	*Overall sample*	*S-TOFHLA categories*	
		Low HL (n = 64)	High HL (n = 158)
*Age (years)*	60.6 ± 10.7	63.9 ± 12.8	60.3 ± 9.6
*Male (n)*	130 (58.6)	44 (68.8)	86 (54.4)
*BMI (kg/m^2^)* *	33.6 ± 8.1	34.4 ± 8.9	33.2 ± 7.7
*Years of formal education* *	11.3 ± 3.3	10.1 ± 2.5	11.7 ± 3.5
*Duration of diabetes (years)*	18.0 ± 13.4	20.1 ± 14.0	17.1 ± 13.1
*Insulin Therapy*	173 (77.9)	56 (87.5)	117 (74.1)
*Employment status*			
* Employed full-time*	45 (20.3)	6 (13.3)	39 (86.7)
* Employed part-time*	13 (5.9)	3 (23.1)	10 (76.9)
* Not in paid employment*	163 (73.8)	55 (33.7)	108 (66.3)
*Household Income*			
* <$49,999*	148 (66.7)	52 (81.3)	96 (60.8)
* $50,000-$99,999*	27 (12.2)	3 (4.7)	24 (15.2)
* >$100,000*	18 (8.1)	1 (1.6)	17 (10.8)
* Rather not say*	29 (13.1)	8 (12.5)	21 (13.3)
*Current or ex-smoker (n)*	129 (58.1)	39 (60.9)	91 (57.6)
*Never-smoker (n)*	92 (41.4)	25 (39.1)	67 (42.4)
*PHQ-9 (Depression) (/9)* *	7.2 ± 6.3	8.3 ± 6.8	6.8 ± 6.0
*Diabetes Distress (/6)*	1.7 ± 0.8	1.7 ± 0.7	1.8 ± 0.8
*MOCA (Cognition) (/30)*	25.7 ± 3.5	22.9 ± 3.9	26.8 ± 2.6
*Cognitive impairment, MOCA (<26/30)*	88 (39.6)	44 (68.8)	44 (27.8)
*HLQ Domains*			
*Domain 1: Feeling understood and supported by health professionals*	3.26 (0.52)		
*Domain 2: Having sufficient information to manage health*	3.06 (0.45)		
*Domain 3: Actively managing my health*	2.81 (0.50)		
*Domain 4: Social support for health*	2.97 (0.60)		
*Domain 5: Appraisal of health information*	2.83 (0.57)		
*Domain 6: Ability to actively engage with health care professionals*	4.07 (0.69)		
*Domain 7: Navigating the healthcare system*	3.94 (0.64)		
*Domain 8: Ability to find good health information*	3.84 (0.76)		
*Domain 9: Understanding health information well enough to know what to do*	4.00 (0.68)		

*All n = 222, except where noted; BMI, PHQ-9, Diabetes Distress n = 221, Years of formal education n = 220, Employment status n = 221

S-TOFHLA: Short form Test of Functional Health Literacy in Adults, scored /36. Low HL: ≤31/36, High HL ≥32/36. HLQ Domains 1–5 have a score range of 1–4; domains 6–9 have a score range of 1–5.

BMI: body mass index; MOCA: Montreal Cognitive Assessment; PHQ-9: Patient Health Questionnaire (9 items)

Values are mean ± SD or n(%) where stated.

Participants were predominantly male (58.6%) with mean (SD) age of 60.6 (10.7) years and 11.3 (3.3) years of formal education. The average duration of diabetes was 18 years and 77.9% were insulin treated. The mean BMI was 33.6 (8.1) kg/m^2^. Mean(SD) cognition scores (on the MOCA) was 25.7 (3.5) (possible score range 0–30) with 88 (39.6%) individuals having cognitive impairment (MOCA <26/30).

The median S-TOFHLA score was 34 (IQR 32–36). There were 204 individuals assessed as having adequate, 6 with marginal and 12 with inadequate functional health literacy according to previously published categories [19], and 158 participants with high and 64 with low functional health literacy according to the dichotomized S-TOFHLA categories described above. For individual HLQ domains, participants recorded the highest scores for domain 1: feeling understood and supported by health professionals, with a mean(SD) score of 3.26 (0.52) with 4 being the maximum possible score. Lowest scores were reported on domain 3: Actively managing my health, with a mean(SD) score of 2.81 (0.50) (Table 1).

Participants with low functional health literacy were older, had fewer years of formal education, were experiencing higher levels of diabetes-related distress, and scored lower on the MOCA than those with adequate health literacy (Table 1).

### Associations of demographic variables with health literacy

The estimated associations of risk factors with low functional health literacy (measured by S-TOFHLA) are given in Table 2. The odds of having low functional health literacy were higher with older age (OR 1.03, 95% CI 1.00, 1.06) and lower with better cognition (OR 0.69, 95% CI 0.62, 0.77), greater years of formal education (OR 0.83, 95% CI 0.73, 0.93), and higher household income (OR 0.11, 95% CI 0.01, 0.56 for individuals earning >$100,000 per annum) in univariable analyses. With the exception of age and household income, these associations persisted in multivariable analysis (Table 2). Higher scores on the depression questionnaire (PHQ-9), diabetes distress and smoking status were not associated with functional health literacy in univariable analyses.

**Table 2:** Estimated odds ratios and 95% confidence intervals (CI) from logistic regression models of functional health literacy and patient risk factors.

*Variable*	*Univariable logistic regression*		*Multivariable logistic regression*	
	Odds ratio	95% CI	Odds ratio*	95% CI
*Age (years)*	**1.03**	**1.00, 1.06**	1.003	0.97, 1.04
*Years of Formal Education*	**0.83**	**0.73, 0.93**	**0.88**	**0.76, 0.99**
*Cognition (MOCA)*	**0.69**	**0.62, 0.77**	**0.71**	**0.63, 0.79**
*Gender (Male)*	1.77	0.96, 3.33	1.73	0.85, 3.66
*Diabetes Distress*	0.95	0.64, 1.36	Not included in final multivariable model	
*Depression (PHQ-9)*	1.04	0.99, 1.08		
*Household Income*				
*<$49,999*	Reference category			
*$50,000-$99,999*	**0.24**	**0.06, 0.74**		
*>$100,000*	**0.11**	**0.01, 0.56**		
*Rather not say*	0.75	0.29, 1.76		
*Current or ex smoker*	0.89	0.49, 1.61		

*Logistic regression modelling; odds ratio indicates transition from a higher functional health literacy to lower functional health literacy category

Bold denotes p<0.05

MOCA: Montreal Cognitive Assessment

PHQ-9: Patient Health Questionnaire (9 items).

Table 3 shows the estimated associations of risk factors with different domains on the HLQ in both univariable and multivariable analysis. Diabetes distress was associated with the most HLQ domains (five out of nine multivariable analyses), these being domain 2 *(having sufficient information to manage my health)* (β = -0.14, 95% CI -0.23, -0.06), domain 4 (*social support for health*) (β = -0.18, 95% CI = -0.3, -0.07), domain 6 (*ability to actively engage with healthcare providers*) (β = -0.16, 95% CI -0.29, -0.02), domain 7 (*navigating the healthcare system*) (β = -0.18, 95% CI -0.3, -0.05) and domain 8 (*ability to find good health information*) (β = -0.15, 95% CI -0.3, -0.02). Educational attainment was positively associated with four domains (domain 2 *(having sufficient information to manage my health)* (β = 0.03, 95% CI 0.02, 0.05), domain 3 (*actively managing my health*) (β = 0.04, 95% CI 0.02, 0.06), domain 5 (*appraisal of health information*) (β = 0.04, 95% CI 0.02, 0.06) and domain 8 (*ability to find good health information*) (β = 0.04, 95% CI 0.01, 0.07).

**Table 3:** Models of the association between mean of each HLQ domain score and demographic characteristics in univariable and multivariable linear regression.

		Domain 1	Domain 2	Domain 3	Domain 4	Domain 5	Domain 6	Domain 7	Domain 8	Domain 9
**Age (years)**										
**Univariable**		-0.002 (-0.008, 0.005)	-0.003 (-0.009, 0.002)	-0.002 (-0.008, 0.004)	-0.003 (-0.01, 0.005)	-0.01 (-0.02, 0.004)	0.002 (-0.006, 0.01)	0.0008 (-0.007, 0.009)	-0.01 (-0.02, -0.004)	**-0.009 (-0.02, -0.0002)**
**Multivariable**		-0.001 (-0.01, 0.006)	-0.003 (-0.009, 0.002)	-0.002 (-0.01, 0.004)	-0.004 (-0.01, 0.003)	**-0.01 (-0.02, -0.002)**	0.002 (-0.007, 0.01)	0.0001 (-0.008, 0.008)	**-0.01 (-0.02, -0.003)**	-0.007 (-0.01, 0.002)
**Gender (Male)**										
**Univariable**		-0.06 (-0.2, 0.08)	-0.02 (-0.14, 0.10)	-0.006 (-0.13, 0.14)	-0.12 (-0.28, 0.04)	-0.13 (-0.28, 0.02)	-0.07 (-0.25, 0.12)	-0.08 (-0.26, 0.09)	-0.16 (-0.35, -0.05)	**-0.28 (-0.46, -0.09)**
**Multivariable**		-0.07 (-0.21, 0.07)	-0.04 (-0.16, 0.07)	-0.03 (-0.16, 0.09)	-0.15 (-0.30, 0.005)	-0.13 (-0.28, 0.02)	-0.09 (-0.27, 0.1)	-0.1 (-0.27, 0.07)	-0.15 (-0.35, 0.04)	**-0.28 (-0.45, -0.1)**
**Years of formal education**										
**Univariable**		**0.02 (0.002, 0.04)**	**0.03 (0.02, 0.05)**	**0.04 (0.02, 0.06)**	0.01 (-0.01, 0.04)	**0.04 (0.02, 0.06)**	0.01 (-0.01, 0.04)	0.01 (-0.01, 0.04)	0.05 (0.02, 0.08)	**0.03 (0.01, 0.07)**
**Multivariable**		0.02 (-0.001, 0.04)	**0.03 (0.02, 0.05)**	**0.04 (0.02, 0.06)**	0.003 (-0.02, 0.03)	**0.04 (0.02, 0.06)**	0.01 (-0.02, 0.04)	0.01 (-0.01, 0.04)	**0.04 (0.01, 0.07)**	0.02 (-0.005, 0.05)
**BMI (kg/m^2^)**										
**Univariable**		-0.002 (-0.01, 0.006)	-0.001 (-0.009, 0.006)	-0.01 (-0.02, -0.005)	-0.007 (-0.02, 0.003)	0.004 (-0.005, 0.01)	0.002 (-0.009, 0.01)	0.0008 (-0.01, 0.01)	0.003 (-0.01, 0.02)	-0.004 (-0.01, 0.008)
**Multivariable**		-	-	-	-	-	-	-	-	-
**MOCA (/30)**										
**Univariable**		0.01 (-0.005, 0.03)	0.003 (-0.01, 0.02)	-0.004 (-0.02, 0.02)	0.005 (-0.02, 0.003)	0.02 (-0.003, 0.04)	0.01 (-0.01, 0.04)	0.008 (-0.02, 0.03)	**0.04 (0.008, 0.07)**	**0.04 (0.01, 0.07)**
**Multivariable**		0.007 (-0.01, 0.03)	-0.009 (-0.03, 0.009)	-0.02 (-0.04, 0.002)	-	0.002 (-0.02, 0.02)	-	0.001 (-0.02, 0.03)	0.02 (-0.01, 0.04)	0.02 (-0.002, 0.05)
**PHQ-9 (/9)**										
**Univariable**		-0.007 (-0.02, 0.004)	**-0.01 (-0.02, -0.004)**	**-0.02 (-0.03, -0.01)**	**-0.02 (-0.04, -0.01)**	-0.003 (-0.02, 0.009)	**-0.02 (-0.04, -0.007)**	**-0.02 (-0.03, -0.005)**	**-0.02 (-0.04, -0.005)**	**-0.02 (-0.04, -0.001)**
**Multivariable**		-	0.0001 (-0.01, 0.01)	**-0.02 (-0.03, -0.004)**	-0.01 (-0.03, 0.002)	0.007 (-0.007, 0.02)	-0.01 (-0.03, 0.008)	-0.005 (-0.02, 0.01)	-0.007 (-0.03, 0.01)	-0.01 (-0.03, 0.003)
**Diabetes Distress (/6)**										
**Univariable**		-0.08 (-0.16, 0.009)	**-0.13 (-0.21, -0.06)**	**-0.12 (-0.21, -0.04)**	**-0.22 (-0.32, -0.13)**	-0.05 (-0.14, 0.05)	-0.19 (-0.30, -0.08)	**-0.19 (-0.30, -0.09)**	**-0.16 (-0.28, -0.03)**	**-0.16 (-0.27, -0.05)**
**Multivariable**		-0.08 (-0.17, 0.01)	**-0.14 (-0.23, -0.06)**	-0.07 (-0.16, 0.03)	**-0.18 (-0.30, -0.07)**	-0.1 (-0.21, 0.008)	**-0.16 (-0.29, -0.02)**	**-0.18 (-0.3, -0.05)**	**-0.15 (-0.3, -0.02)**	-0.12 (-0.25, 0.003)
**Household Income**										
**<$49,999**	Univariable	Reference category								
**$50,000-$99,999**		0.1 (-0.12, 0.31)	0.10 (-0.08, 0.29)	0.03 (-0.17, 0.24)	**0.26 (0.01, 0.50)**	0.06 (-0.17, 0.30)	0.23 (-0.05, 0.51)	0.14 (-0.13, 0.40)	0.28 (-0.02, 0.59)	0.20 (-0.09, 0.48)
**>$100,000**		0.07 (-0.19, 0.33)	0.11 (-0.11, 0.34)	0.19 (-0.05, 0.44)	0.21 (-0.08, 0.50)	0.23 (-0.06, 0.51)	0.18 (-0.16, 0.52)	0.14 (-0.18, 0.45)	0.52 (0.15, 0.88)	0.33 (-0.01, 0.66)
**Rather not say**		0.06 (-0.15, 0.27)	0.10 (-0.08, 0.29)	-0.12 (-0.31, 0.08)	0.15 (-0.09, 0.39)	0.04 (-0.19, 0.27)	0.15 (-0.12, 0.42)	0.09 (-0.16, 0.35)	0.21 (-0.09, 0.51)	-0.008 (-0.28, 0.26)

Presented as betas (95% CI);—denotes not included in model

*Linear regression modelling; models additionally adjusted for household income and BMI. Household Income not included in multivariable models.

Bold denotes p<0.05

BMI: Body Mass Index; MOCA, Montreal Cognitive Assessment (maximum score 30); PHQ-9, Patient Health Questionnaire (nine items, maximum score 27);

HLQ domains: 1: Feeling understood and supported by healthcare providers; 2: Having sufficient information to manage my health; 3: Actively managing my health; 4: Social support for health; 5: Appraisal of health information; 6: Ability to actively engage with healthcare providers; 7: Navigating the healthcare system; 8: Ability to find good health information; 9: Understanding health information well enough to know what to do

In multivariable analysis, age was negatively associated with domains 5 (*appraisal of health information*) (β = -0.01, 05% CI -0.02, -0.002) and domain 8 (*ability to find good health information*) (β -0.01, 95% CI = -0.02, -0.003). Being male was negatively associated with domain 9 (*understanding health information well enough to know what to do)* (β = 0.28, 95% CI = -0.45, -0.1). Scores on the PHQ-9 were negatively associated with domain 3 (*actively managing my health*) only (β = -0.02, 95% CI -0.03, -0.004) in multivariable analysis. The only predictor of scores on domain 1 (*feeling understood and supported by healthcare providers*) was years of formal education (β = 0.02, 95% CI 0.002, 0.04) in univariable analysis only and cognition was associated with domains 8 (*ability to find good health information*) and 9 (*understanding health information well enough to know what to do*) also only in univariable analyses.

## Discussion

This study is the first to examine predictors of both functional and the full range of health literacy domains amongst individuals with diabetes. There were different predictors of functional health literacy and other health literacy domains. Cognition and poorer educational attainment were associated with poor functional health literacy whereas diabetes-related distress and lower educational attainment were associated with lower scores in multiple health literacy domains. Every one-unit increase in MOCA score was associated with an 29% reduction in odds of having low functional health literacy, and each additional year of formal education was associated with a 12% reduction in odds of having low functional health literacy. Higher levels of diabetes-related distress was associated with poorer HLQ scores in the highest number of domains (five), whereas higher educational attainment was positively associated with scores on four HLQ domains. Overall, people with cognitive deficits, poorer education levels and individuals experiencing high levels of diabetes related distress are at risk of poorer health literacy. Being aware that these groups may encounter significant barriers when attempting to access healthcare may assist healthcare providers and policy makers to improve their approaches to delivering diabetes related education. Addressing such barriers in health policy and clinical practice is crucial to delivering patient-centered care and shared decision making.

Cognitive functioning may be crucial for functional health literacy in people with diabetes. It has been proposed that measures of health literacy are actually crude assessments of general cognitive abilities such as fluid (learning and application of new information) and crystallized abilities (background knowledge) [20]. Similarly, health literacy fundamentals such as reading and numeracy, are considered essential cognitive skills required to navigate healthcare systems [21]. Cognitive decline is an important issue in diabetes; type 2 diabetes has been associated with cognitive decline (executive ability and memory) [22], and a 50% increased risk of having two or more cognitive deficits that interfere with daily activities [23]. The 29% increase in the odds of having poorer health literacy with every unit decrease in MOCA scores in our study is consistent with previous findings of a strong relationship between functional health literacy and cognition [12]. Current guidelines recommend simplifying medication regimens and tailored glycemic targets for people with diabetes and cognitive decline [24], and these principles of simplified communication and healthcare goals should be applied to people with cognitive deficits and at risk of low functional health literacy.

Diabetes distress may adversely affect health literacy across multiple domains of having sufficient information to manage health; social support for health; the ability to actively engage with healthcare professionals; navigating the healthcare system; and the ability to find good health information. This mirrors recent findings of better psychological wellbeing being associated with higher levels of health literacy in patients with COPD [25]. Diabetes distress represents the psychological and emotional distress of being overwhelmed by the constant and challenging demands of adhering to diabetes self-management requirements and is prevalent amongst the diabetes community [18]. Living with diabetes distress can negatively affect self-care, adherence to medication, is detrimental to overall glycemic control [26] and reduces the positive impact adequate health literacy has on diabetes self-care and diabetes self-efficacy [27]. Our findings suggest that people living with diabetes distress may struggle due to having inadequate health information, and being unable to source this themselves [14]. Consistent with our findings, they are also more likely to feel alone and unsupported, are passive in their approach to healthcare and be unable to advocate for their own health [14]. This could be addressed in a number of ways. At an individual level, consideration could be given to psychological interventions addressing diabetes distress. These can improve diabetes-related self-efficacy and short-term HbA1c but there is insufficient evidence to determine whether they can similarly improve diabetes-related complications [28] or if reducing diabetes distress will improve health literacy itself. This requires further investigation. However, there is also an important shift away from the traditional view of health literacy as an individual deficit to it being conceptualized as individuals, their communities, and healthcare providers working in partnership to ensure healthcare requirements of all individuals are met [29–31] Identifying people with diabetes distress and utilizing their carers or support networks to advocate for them and assist with obtaining information is thus also an important approach to mitigating some of the effects of both inadequate health literacy and diabetes distress itself that requires further examination.

Educational attainment has been reported to be associated with poorer health literacy [10, 11], and our findings are consistent with this, with each additional year of formal education attained reducing the odds of poorer functional health literacy by 12%. Furthermore, poorer educational attainment was associated with lower scores in four HLQ domains. Of these, three relate to having sufficient information to manage health; appraisal of health information; and ability to find good health information, and the fourth relates to being able to actively manage health. This is unsurprising; education is an established social determinant of health and plays a key role in empowering better health choice [32]. Improving educational outcomes in a given community may be one important way to improve health literacy at a community level. Lower educational attainment also helps identify individuals at risk of poor health literacy in clinical practice. It is imperative for clinicians and policymakers to work in partnership, and to understand strategies which may facilitate better ways for people with poor educational attainment to obtain and retain health information, subsequently empowering them to achieve better health outcomes.

A major strength of this study is the inclusion of a wide range of sociodemographic and psychometric measures, including cognition, depression and diabetes distress. Another is the use of two health literacy measures as previously discussed, and the description of health literacy deficits amongst people with diabetes at risk of foot disease using the HLQ. This more comprehensive information about health literacy will enable clinicians and policy makers to better understand areas of health literacy in their communities that need to be addressed whilst supporting and empowering people to better manage their diabetes care. Our study also has limitations. The distribution of STOFHLA scores was skewed, with 91.9% of participants having adequate health literacy according to previously published cut-offs for the S-TOFHLA. This prevented the use of those published cut-offs in our analyses. However, though the published cut-offs are commonly used, they were derived from a single study of patients from two US public hospitals [19], and their criterion validity has not been established. The associations between S-TOFHLA categories and education and cognition in our study suggest that the cut-offs we used are appropriate for the distribution of scores in our population. The response rate of 54%, and recruitment from a single tertiary outpatient clinic in Australia may reduce the generalizability of our findings, particularly to people with diabetes being managed outside of a tertiary care setting. However, to our knowledge it is the first study of its kind to measure health literacy amongst people with diabetes in an outpatient setting in Australia and the first to measure both functional and overall health literacy in people with diabetes globally so it nonetheless makes a substantial contribution to the knowledge in this area. Furthermore, the rates of inadequate functional health literacy according to published cut-offs in our study (5.4%) were similar to those of another Australian community based study [33], suggesting that health literacy levels between community and outpatient settings may not differ greatly.

## Conclusions

In conclusion, poorer cognition and poorer educational attainment may help identify individuals with low functional health literacy amongst people living with diabetes. Educational attainment, diabetes distress and older age may identify those with lower health literacy across multiple domains. Clinicians and policy makers should be attuned to these factors when communicating with people with diabetes and in designing healthcare systems to be more health-literacy friendly in order to improve diabetes outcomes.
